# Differential expression of seven conserved microRNAs in response to abiotic stress and their regulatory network in *Helianthus annuus*

**DOI:** 10.3389/fpls.2015.00741

**Published:** 2015-09-17

**Authors:** Reyhaneh Ebrahimi Khaksefidi, Shirin Mirlohi, Fahimeh Khalaji, Zahra Fakhari, Behrouz Shiran, Hossein Fallahi, Fariba Rafiei, Hikmet Budak, Esmaeil Ebrahimie

**Affiliations:** ^1^Department of Plant Breeding and Biotechnology, Faculty of Agriculture, Shahrekord UniversityShahrekord, Iran; ^2^Department of Agricultural Biotechnology, Institute of Biotechnology, Shahrekord UniversityShahrekord, Iran; ^3^Department of Biology, School of Sciences, Razi UniversityKermanshah, Iran; ^4^Biological Sciences and Bioengineering Program, Faculty of Engineering and Natural Sciences, Sabanci UniversityIstanbul, Turkey; ^5^Faculty of Agriculture, Institute of Biotechnology, Shiraz UniversityShiraz, Iran; ^6^Department of Genetics and Evolution, School of Biological Sciences, University of AdelaideAdelaide, SA, Australia; ^7^School of Biological Sciences, Faculty of Science and Engineering, Flinders UniversityAdelaide, Australia

**Keywords:** abiotic stress, miRNA, sunflower (*Helianthus annuus*), regulatory network

## Abstract

Biotic and abiotic stresses affect plant development and production through alternation of the gene expression pattern. Gene expression itself is under the control of different regulators such as miRNAs and transcription factors (TFs). MiRNAs are known to play important roles in regulation of stress responses via interacting with their target mRNAs. Here, for the first time, seven conserved miRNAs, associated with drought, heat, salt and cadmium stresses were characterized in sunflower. The expression profiles of miRNAs and their targets were comparatively analyzed between leaves and roots of plants grown under the mentioned stress conditions. Gene ontology analysis of target genes revealed that they are involved in several important pathways such as auxin and ethylene signaling, RNA mediated silencing and DNA methylation processes. Gene regulatory network highlighted the existence of cross-talks between these stress-responsive miRNAs and the other stress responsive genes in sunflower. Based on network analysis, we suggest that some of these miRNAs in sunflower such as *miR172* and *miR403* may play critical roles in epigenetic responses to stress. It seems that depending on the stress type, theses miRNAs target several pathways and cellular processes to help sunflower to cope with drought, heat, salt and cadmium stress conditions in a tissue-associated manner.

## Introduction

Abiotic and biotic stresses impose challenging physiological hurdles to plants. As a response to adverse environmental conditions, plants re-program their cellular activities through multiple gene regulatory mechanisms including post-transcriptional regulation of gene expression. Transcription factors (TFs) and non-coding RNAs are the two major regulatory elements in functional genomics (Deihimi et al., 2012; Mahdi et al., 2013; Panahi et al., 2013; Chiasson et al., 2014).

Small RNAs, particularly microRNAs (miRNAs), have emerged as key post-transcriptional gene regulatory molecules in plants and animals (Trindade et al., 2010; Giacomelli et al., 2012; Sunkar et al., 2012). MiRNAs are 18–22 nucleotides in length and regulate expression of their target genes by degradation or translational repression (Voinnet, 2009; Sunkar et al., 2012). In particular, miRNAs play important roles in plant responses to abiotic and biotic stresses including cold, salt, heat, dehydration, oxidative and mechanical stresses (Jin et al., 2013; Panahi et al., 2013). For example, some of the genes in *ARF* transcription factor family are targeted by *miR160* and *miR167* through auxin regulation interference (Gutierrez et al., 2009), and seem to be important for the attenuation of plant growth and development under stress conditions (Sunkar et al., 2012). Another microRNA, *miR398*, is associated with response to abiotic and biotic stress condition (Zhu et al., 2011) and its expression is up-regulated in response to water deficit in *Medicago truncatula* (Trindade et al., 2010). *MiR403* controls the expression of *AGO2* (Allen et al., 2005), which is known to have an antiviral role (Harvey et al., 2011). Srivastava et al. (2013) reported that *miR426* is up-regulated in response to arsenic stress in *Brassica juncea*. In another study, *miR842* expression levels were observed to be up-regulated in *Arabidopsis* (Moldovan et al., 2010). These are handful examples of miRNAs that are differentially expressed in response to various stress conditions.

Sunflower (*Helianthus annuus* L.) is one of the most important oilseed crops and is resistant to various abiotic stresses, due to its metabolic, physiological and morphological adaptation strategies. This crop is of special interest for its adaptation to high temperatures, limited water availability, high salinity and heavy metal concentrations in the soil (Merah et al., 2012). Using bioinformatics methods, previous studies have identified and reported several miRNAs in sunflower. A number of these were experimentally verified (Barozai et al., 2012; Monavar Feshani et al., 2012). However, their expression patterns under different stress conditions have not been studied.

We have examined the roles of seven of these sunflower miRNAs in response to different abiotic stresses including drought, high temperature, salinity and cadmium. Seven mentioned miRNAs, *miR160, miR167, miR172, miR398, miR403, miR426* and *miR842*, play crucial roles in plant growth and show different expression patterns under both biotic and abiotic stress conditions in several plant species. Simultaneously, we analyzed the expression levels of these miRNAs concurrently with their potential targets to corroborate the putative miRNA-target relationship. Based on our results, several gene regulatory networks were constructed to gain a comprehensive overview of the role of these miRNAs in stress responses.

## Materials and methods

### Prediction of miRNA targets

Most plant miRNAs bind to their targets with perfect or near-perfect complementarity (Jones-Rhoades and Bartel, 2004). We have used this characteristic for predicting potential miRNA targets. Sequences of pre-miRNAs were obtained from the public database, miRBase (Kozomara and Griffiths-Jones, 2011). The number of mismatches at complementary sites between miRNA sequence and potential mRNA target were kept below 4 and no gap at the complementary sites was tolerated. The miRNA targets were predicted using psRNAtarget online sever (http://plantgrn.noble.org/psRNATarget/).

### Plant material, growth conditions and stress treatments

Sunflower (*Helianthus annuus*, variety *Sirna*) seeds were surface sterilized and placed in petri dishes containing two layers of damp sterile filter paper. For germination, the seeds were incubated at room temperature for 4 days.

For applying drought stress treatment, five germinated seeds were sown in round pots (10.5 × 13.5 cm, D × H; five plants/pot) filled with soil (¾ river sand and ¼ soil). Plants were grown in the glasshouse under the controlled condition (16 h of light, 24°C; 8 h of dark, 15°C) and well watered.

For heat, salinity and cadmium stress treatments, five seedlings were grown in a hydroponic system. The system contained aerated Hoagland solutions, pH 5.8 (Hoagland and Arnon, 1950) in plastic pots (1.5 liters volume). One-week-old plantlets were planted in ¼ strength Hoagland's nutrient for 1 more week to immerse the growing roots in the nutrient solution. Plantlets were then transferred to half-strength nutrient solution and grown until 6-leave stage (about 4 weeks). Four weeks old plantlets were then submitted to different stresses as specified in the following sections.

### Drought stress

Four weeks old plantlets (6 leaf stage) of sunflower were subjected to water stress by withholding water for 12, 24 and 48 h. Relative water content (RWC) was measured in leaves to determine the plant water status following Faatsky method (Čatský, 1960).

### Heat stress

Four weeks old plantlets in ½ strength Hoagland solution were transferred to humidity growth chamber (Memmert, Germany) with 70% relative humidity and maintained at 42 ± 1°C for 1.5, 3 and 6 h (Senthil-Kumar et al., 2003; Mashkina et al., 2010; Mangelsen et al., 2011). We selected stress-related *HSP70*-related protein gene (Gene Bank ID: AAB57695.1) to investigate the effect of heat stress in root tissue of sunflower.

### Salt stress

Plantlets were moved into solution contained either 75 or 150 mM of NaCl (Di Caterina et al., 2007). Leaf and root samples from stressed plants were collected 24 h later alongside control samples. Potassium to sodium ratio was measured as criteria for examining different salinity stress levels.

### Cadmium stress

Plantlets were transferred into containers with either 5 or 20 mg/L of CdCl_2_ (Niu et al., 2007). Stress and control samples were collected after 24 h from leaf and root tissue and immediately frozen in liquid nitrogen before they stored at −80°C. Cadmium content was measured as an indicative of stress.

### Total RNA isolation from leaves and roots

Total RNAs were extracted from sunflower root and leaf tissues using TRIZOL Reagent (Invitrogen, USA) according to the manufacturer's instructions. The extractions were performed separately for two independent biological replicates. Equal amount of total RNA was subsequently pooled for each sample based on their concentration. The quantity and quality of purified RNAs were assessed with a Biophotometer spectrophotometer (Eppendorf, Germany) and the integrity was evaluated by conducting a denaturing agarose gel electrophoresis. All RNA samples were stored at −80°C until further processing.

### Stem-loop reverse transcription

Stem-loop RT primers for *H. annuus miR160, miR167, miR172, miR398, miR403, miR426* and *miR842* were designed according to Varkonyi-Gasic et al. (2007) (Table S1). The miRNA specific RT reactions were carried out using prime script RT reagent kit (Cat. #RR037A, TAKARA, Japan). The miRNA stem–loop reverse transcription was accomplished according to Varkonyi-Gasic et al. (2007) with minor modifications at cDNA synthesis, using 200 ng of total RNA sample of treated leaf and root tissues (1 μL), 1 μL of 1 μM miRNA primer RT and 5.5 μL nuclease-free water. The mix was incubated at 65°C for 5 min followed by incubation on ice for 2 min. Two microliters of Primescript buffer (5×) and 0.5 μL Primescript RT enzyme mix were added to each tube and the RT reaction was performed for 30 min at 16°C followed by 60 cycles at 30°C for 30 s, 42°C for 30 s, 50°C for 1 s and terminated by incubation at 85°C for 5 min. Control reactions were performed with no RT primer and no RNA samples.

### Quantitative real-time PCR

To measure and compare the expression levels of the selected *H. annuus* miRNAs in root and leaf tissues under different stress treatments, RT-qPCR was conducted using SYBR *Premix Ex Taq* II (Cat. #RR820A, TAKARA) in a Rotor-GeneQ Real-Time PCR system (Qiagen).

For RT-qPCR analysis, 6 μL SYBR *Premix Ex Taq* II (2×), 1 μL (10 pmol) each of forward and reverse primers, 3 μL nuclease-free water and 1 μL RT stem-loop cDNA product were mixed. Forward primers were specifically designed for each miRNA, and 5′-GTGCAGGGTCCGAGGT-3′ sequence was used as the universal reverse primer (Varkonyi-Gasic et al., 2007) (Table S1). The RT-qPCR reactions were performed using following conditions; initial denaturation at 95°C for 30 s, followed by 40 cycles at 95°C for 5 s and 60°C for 20 s. The melting curves were generated during denaturation step at 95°C followed by the cooling of PCR products at 50°C and the fluorescence signals were collected continuously from 50 to 95°C as the temperature increased at 0.2°C per second. All reactions were repeated four times. For each condition, the RT-qPCR experiments were run as pooled biological duplicates and expression levels were normalized according to the previous studies (Schmittgen and Livak, 2008). Relative fold changes in miRNA expression were calculated using the comparative Ct (2^−ΔΔCt^) method with *18S rRNA* as the endogenous control (Schmittgen and Livak, 2008). The miRNAs clustering analysis, based on their relative expression, and Pearson correlation between expression miRNAs and target genes in each stress were performed using Minitab 16.0. The heat map of expression was visualized using R 3.0 package.

### Analysis of miRNAs target expression with RT-qPCR

To determine the expression of predicted miRNA targets and possible discovery of novel miRNA target genes under the drought, heat, salinity and cadmium stresses in sunflower, expression levels of miRNA-related target genes were measured with quantitative real-time PCR. ESTs for target quantification analysis were selected based on two criteria: (1) protein found in BLAST search should be a previously published target of the related miRNA in other plant species. (2) The EST should have a possible protein-coding ORF that can allow us to design RT-qPCR primers for the conserved 3′ UTR region. RT-qPCR primers for these selected genes (Table S1) were designed using Primer3 according to following criteria: (1) Primers 3′ self-complementary = 0. (2) Primer annealing temperatures = 62 ± 0–3°C. (3) Product size limit for primer pairs = 150 bp.

Total cDNAs were synthesized from 1 μg RNA using RevertAid™ First Strand cDNA Synthesis Kit (Fermentas) according to the manufacturer's instructions. Further expression analysis was performed for all miRNA targets using the same batch of RNA samples for miRNA RT-qPCR assay. One microliter of this cDNA was amplified with 0.6 μM of specific primers in a total of 10 μl volume using SYBER *Premix Ex Taq* II (Cat. #RR820A, TAKARA) with Rotor-GeneQ Real-Time PCR system (Qiagen). The quantification was performed using *18S rRNA* (Gene ID: 18250984, forward: TTCAGACTGTGAAACTGCGAATGG /reverse: TCATCGCAGCAACGGGCAAA) as a normalizer and four independent PCR results with acceptable efficiency (1.8–2.2) were averaged. Specified RT-qPCR thermal setup was adjusted as follows: pre-denaturation step at 95°C for 1 min, followed by 40 cycles of 95°C for 30 s, 60°C for 1 min. The melting curves were generated as mentioned for miRNAs.

### Network analysis of miRNAs

To construct miRNAs and target genes interaction network, RESNET Plant database of Pathway Studio software v.9 (Elsevier) was used. This database includes new aliases for genes in the model and non-model plant species including barely, corn, tomato, potato and tobacco and collects data through MedScan (text mining tool) to extract functional relationships between miRNAs, proteins, stresses and cellular processes (Nikitin et al., 2003; Alimohammadi et al., 2013; Ebrahimie et al., 2014). In addition to adding the result of this study to prediction database of RESNET database, this database was also updated by MedScan, especially from literature on miRNAs/target genes and drought, heat, salt and cadmium stress conditions before network construction. To predict the interaction networks, the software makes different groups of miRNAs and finds the relations between a protein and its group using algorithms such as Fisher's Exact Test (Alanazi et al., 2013; Ebrahimie et al., 2014).

Network constructed by union selected and physical interaction algorithms were used to make statistical subnetworks based on miRNAs and their target genes. Also, GO (gene ontology) analysis was performed through DAVID Functional Annotation web-tool (https://david.ncifcrf.gov/) and separate tables were produced for biological process, molecular function and cellular component categories. MiRNAs and putative target genes are marked with yellow circles (**Figures 6, 7**).

## Results

In this study, expression levels of seven miRNAs including *miR160, miR167, miR172, miR398, miR403, miR426* and *miR842* and their targets were investigated in response to four abiotic stress conditions including drought, heat, salt and cadmium stresses. In addition, the impacts of these stresses on the physiological and molecular characteristics of plants were evaluated by measuring the changes in RWC, expression of *HSP70*-related protein, sodium, potassium and cadmium concentrations in different tissues of plant, grown under stress (Supplementary 1). The expression level of *HSP70*-related protein up-regulated over time compared to control in root tissue with the highest level at 6 h (Table S2). Moreover, alteration of sodium, potassium and cadmium concentration were remarkably higher in root tissue compared to leaf, in particular in later stage of stress (Table S3A). The average RWC of plants in response to drought stress showed slight decline at 12 and 24 h, and it started to reduce sharply subsequently (Table S3B). The results indicated that plants were significantly affected by stress exposure and were able to initiate stress related responses.

### Prediction and determination of *H. annuus* miRNA targets

The mRNA targets of these miRNAs (Table 1) were predicted via psRNAtarget database using selected miRNAs as queries. Targets of *miR167* and *miR403* were previously reported in the other plant species (Table 1). We predicted and analyzed new targets for *miR160, miR398, miR426* and *miR842* for the first time in sunflower. Protein sequence analysis of the new putative *Helianthus miR160* target, *QHG18J04.yg.ab1* showed the presence of ARF, phosphorylase and kinase domains (data not shown) in this protein. Interestingly, *COX5b*, which was previously introduced as *miR398* target in *Arabidopsis*, was identified as a predicted target for *miR172* in sunflower.

**Table 1:** **Putative ***Helianthus annuus*** stress-inducible miRNA target genes**.

**miRNA**	**miRNA sequence**	**Predicted targets**	**Accession number**
miR160g	UGCCUGGCUCCCUGUAUGCCA	QHG18J04.yg.ab1	BU026935
miR167e	UGAAGCUGCCAGCAUGAUCU	Auxin Response Factors6 (ARF6)	839913
miR172d	AGAAUCCUGAUGAUGCUGC	Cytochrome-c oxidase activity (COX5B-2)	844363
miR398	GUGUUCUCAGGUCGCCCC	Glycosyltransferase (NtGT5b)	AB176524.1
miR403	UUAGAUUCACGCACAAACUCG	Argonaute 2 (AGO2)	840016
miR426	CUUUGGAAGUUUGUCCUUAGU	Probable trehalose-phosphate phosphatase 2 (OsTPP2)	AB277360.1
miR842	UCAUGGUCAGAUUCAUCAUCC	(R)-mandelonitrile lyase	AT1G73050

### Expression of miRNAs and putative targets under drought stress

**Figure 1:**
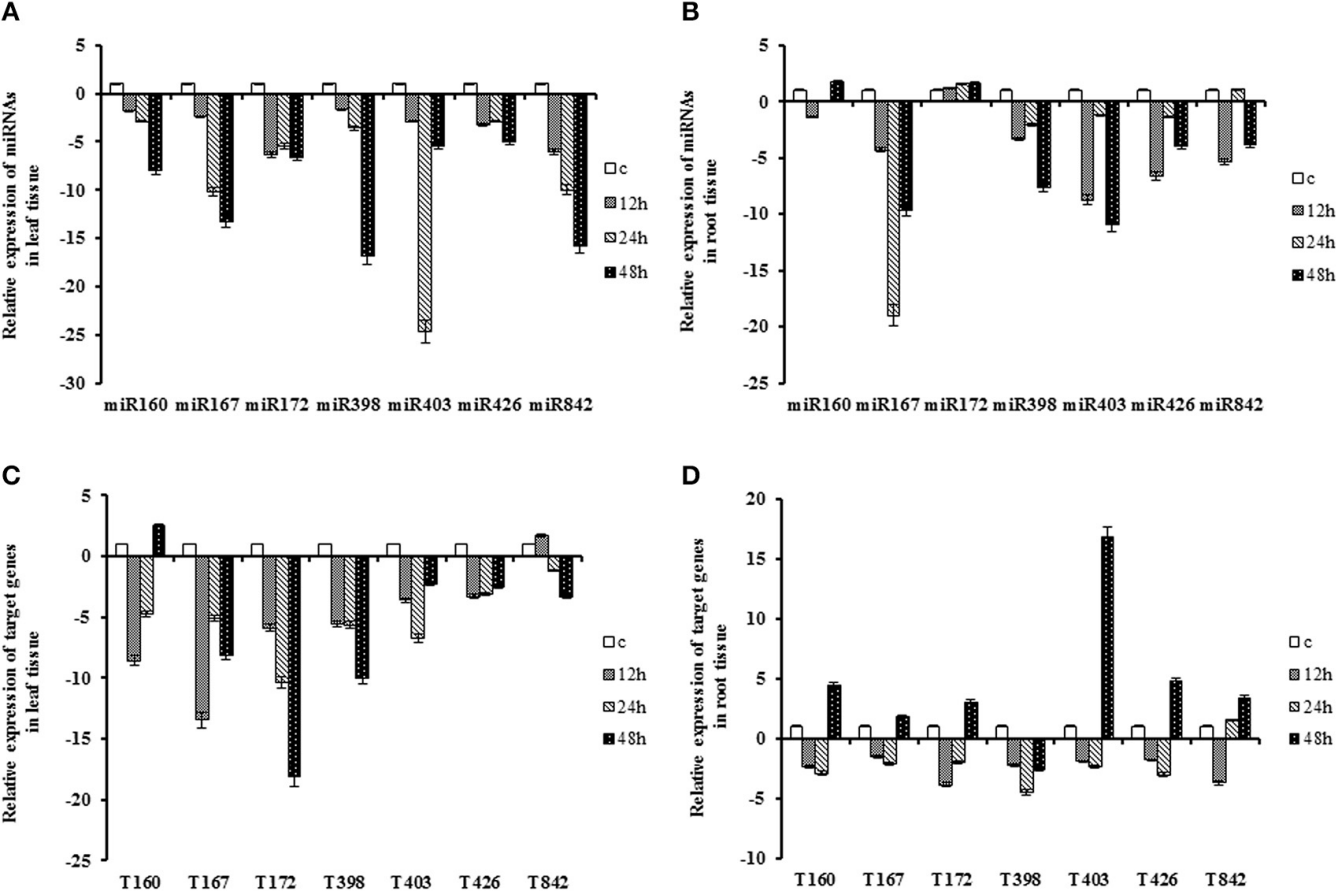
**Expression patterns of miRNAs and their target genes under drought stress**. Sunflower seedling at the six leaf stages grew on soil (¾ river sand and ¼ soil) under normal conditions and leaves and roots of them were harvested as control then they treated by withholding the water for 12, 24 and 48 h. **(A)** Expression patterns of miRNAs in the leaf. **(B)** Expression patterns of miRNAs in the root. **(C)** Expression patterns of target gene in the leaf. **(D)** Expression patterns of target genes in the root.

MiRNAs expression levels were significantly down-regulated (*P* < 0.05) in leaves of plants grown under drought stress with the lowest expression levels were at 48 h (except *miR403*). *MiR172* expression modulation was not significant (*P* < 0.05) in all period of stress in root tissue. The expression patterns of *miR160, miR426* and *miR842* were similar in both tissues, except *miR160* which showed opposite pattern at 48 h after drought stress in root tissue. The miRNAs, *miR167* and *miR398* showed similar trend under drought stress in both tissues. They were down-regulated at all-time points within range of 2- to 19-fold change, whereas expression of *miR167* was slightly up-regulated at 48 h compared to moderate stress in root tissue. *MiR403* showed the opposite pattern in leaf and root tissues at 24 h. Its expression abruptly decreased (24-fold compared to control) in leaf tissue. It, however, exhibited an increasing trend in root tissue while its expression was still lower than control condition (Table 2; Figure 1).

**Table 2:** **Expression of miRNAs and their target genes response to abiotic stress in ***Helianthus annuus*****.

**miRNA/Target name**	**Drought stress**						**Heat stress**						**Salt stress**				**Cadmium stress**			
**Tissue**	**Leaf**			**Root**			**Leaf**			**Root**			**Leaf**		**Root**		**Leaf**		**Root**	
	**12 h**	**24 h**	**48 h**	**12 h**	**24 h**	**48 h**	**1.5 h**	**3 h**	**6 h**	**1.5 h**	**3 h**	**6 h**	**75 mM**	**150 mM**	**75 mM**	**150 mM**	**5 mg**	**20 mg**	**5 mg**	**20 mg**
miR160	↓1.80^**^	↓2.87^*^	↓8.04^*^	↓1.41^ns^	–	↑1.80^**^	↑2.22^**^	↓1.75^**^	↓1.54^*^	↑2.54^*^	↓10.02^**^	↓16.27^**^	↑2.25^**^	↓2.7^**^	↓1.81^*^	↑1.16^ns^	↑2.21^**^	↓2.3538^**^	↑14.35^**^	↑14.85^**^
T160	↓8.57^*^	↓4.75^*^	↑2.54^*^	↓2.40^ns^	↓2.92^ns^	↑4.41^**^	↓6.49^**^	↑7.81^**^	↓1.09^ns^	↓1.36^ns^	↓1.23^ns^	↑2.15^**^	↑4.26^**^	↑1.58^*^	↑1.14^*n*.*s*^	↑1.67^**^	↑2.62^**^	↑5.15^**^	↑32.62^**^	↑47.42^**^
miR167	↓2.41^*^	↓10.19^*^	↓13.22^*^	↓4.28^*^	↓19.02^*^	↓9.67^*^	↑1.56^*^	↓1.39^ns^	↓2.10^**^	↑4.04^**^	↓9.96^**^	↓3.97^**^	↓1.56^*^	↓2.0^**^	↑1.68^*^	↑2.1^**^	↑3.69^**^	↑6.96^**^	↑322.35^**^	↑25.24^**^
T167	↓13.45^*^	↓5.12^*^	↓8.13^*^	↓1.55^*^	↓2.10^*^	↑1.86^**^	↑1.34^ns^	↓1.94^**^	↓2.33^**^	↓1.39^*^	↓39.39^**^	↓57.68^**^	↓1.67^**^	↓1.03^**^	↓1.03^ns^	↑1.23^ns^	↑1.46^ns^	↓1.57^ns^	↑22.51^**^	↑30.75^**^
miR172	↓6.38^*^	↓5.46^*^	↓6.64^*^	↑1.20^ns^	↑1.56^ns^	↑1.62^ns^	–	↓2.43^**^	↓1.20^ns^	↑2.37^**^	↓12.62^**^	↓2.16^**^	↓1.42^ns^	↑1.22^ns^	↓2.0^ns^	↓1.07^ns^	↑1.78^**^	↑1.005^ns^	↑14.27^**^	↑57.38^**^
T172	↓5.85^*^	↓10.37^*^	↓18.06^*^	↓3.86^*^	↓2.0^*^	↑3.03^*^	↑1.36^ns^	↓1.32^ns^	↓1.47^ns^	↑2.92^**^	↓9.18^**^	↓6.84^**^	↓2.44^**^	↓4.76^**^	↓1.70^**^	↓1.17^ns^	↓3.58^**^	↓2.03^**^	↑5.34^**^	↑32.96^**^
miR398	↓1.65^**^	↓3.60^*^	↓16.85^*^	↓3.32^*^	↓2.08^**^	↓7.63^*^	↑3.42^**^	↑1.82^ns^	↑2.68^**^	↑1.65^*^	↓8^**^	↓2.92^**^	↑11.31^**^	↑3.92^*^	↑20.40^**^	↑1.23^ns^	↓1.62^**^	↑1.62^**^	↑385.34^**^	↑123.43^**^
T398	↓5.55^*^	↓5.65^*^	↓10.02^*^	↓2.25^**^	↓4.51^*^	↓2.63^*^	↓10.07^**^	↑3.23^**^	↑1.01^ns^	↓2.28^**^	↑3.17^**^	↓1.28^ns^	↑1.65^*^	↓2.08^**^	↑1.44^ns^	↓2.27^**^	↓16.28^**^	↑1.37^ns^	↓3.83^**^	↑10.93^**^
miR403	↓2.87^*^	↓24.67^*^	↓5.46^*^	↓8.77^*^	↓1.21^ns^	↓10.99^*^	↑3.42^**^	↑1.72^**^	↑1.16^ns^	↑1.20^ns^	↓8.72^**^	↓2.96^**^	↑2.91^**^	↑1.18^ns^	↓1.19^ns^	↓1.41^*^	↑6.28^**^	↑1.02^ns^	↑52.62^**^	↑35.94^**^
T403	↓3.60^*^	↓6.72^*^	↓2.29^ns^	↓1.93^**^	↓2.33^*^	↑16.85^*^	↑2.63^**^	↑1.18^ns^	↓1.12^ns^	↑3.19^**^	↓1.31^ns^	↓1.360^ns^	↑2.83^**^	↓2.51^**^	↑2.46^**^	↑3.25^**^	↑1.07^ns^	↓1.03^ns^	↑22.12^**^	↑65.91^**^
miR426	↓3.19^*^	↓2.87^*^	↓5.06^*^	↓6.61^*^	↓1.41^ns^	↓4.0^*^	↑1.36^ns^	↓1.66^ns^	↑1.28^ns^	↑1.68^**^	↓38.71^**^	↓7.90^**^	↓1.46^**^	↓2.25^**^	↓1.11^ns^	↑9.19^**^	↑1.80^**^	↑1.17^ns^	↑11.65^**^	↑16.77^**^
T426	↓3.30^*^	↓3.08^*^	↓2.46^*^	↓1.83^ns^	↓3.03^*^	↑4.83^*^	↓1.59^**^	↓1.52^*^	↓1.96^**^	↑1.46^**^	↓14.84^**^	↓13.29^**^	↓1.94^**^	↓3.38^**^	↑1.04^ns^	↑2.11^**^	↓2.13^**^	↓2.03^**^	↑10.27^**^	↑14.75^**^
miR842	↓6.06^*^	↓10.02^*^	↓15.81^*^	↓5.37^*^	↑1.05^ns^	↓3.86^*^	↑1.93^ns^	↓1.47^**^	↓1.65^ns^	↓1.49^*^	↓24.11^**^	↓4.72^**^	↑1.19^ns^	↓10.7^**^	↓1.01^ns^	↑3.34^**^	↑1.34^**^	↑2.26^**^	↑10.87^**^	↑4.65^**^
T842	↓1.71^**^	↓1.168^ns^	↓3.30^*^	↓3.73^*^	↑1.53^*^	↑3.40^*^	↑1.09^ns^	↓1.16^ns^	↓5.99^**^	↓1.43^ns^	↓2.37^**^	↓1.67^ns^	↑1.65^*^	↑2.60^**^	↑4.50^**^	↑29.86^**^	↓2.34^**^	↓1.60^ns^	↑7.96^**^	↑32.96^**^

*Sunflower seedling at the six leaf stages grew under normal conditions and leaves and roots of them were harvested as control then they treated by withholding the water for 12, 24 and 48 h. Plants treated at 42 ± 1°C for 1.5, 3, and 6 h. Plant subjected under NaCl treatment in two concentration, 75 and 150 mM. Plants treated with cadmium in 5 and 20 mg/L concentration. Number shows the fold change in relative to control. ↑ The expression is up-regulated. ↓ The expression is down-regulated*.

* *Show that the t-test is significant as p < 0.05*.

** *Show that the t-test is highly significant as p < 0.01*.

ns *show that the t-test is not significant*.

The expression of *QHG18J04.yg.ab1* gene as target for the *miR160*, was down- regulated at 12 h and 24 h (2- to 8-fold), and up-regulated at 48 h in both tissues. Except at 48 h in leaf tissue, correlation of *miR160* and its target showed similar expression pattern over time and tissue. The expression of *ARF6* (target of *miR167*), *COX5b* (target of *miR172*), *AGO2* (target of *miR403*) and *TPP2* (target of *miR426*) displayed similar patterns in both tissues; their expression was induced only in the root tissue at 48 h of drought stress. Interestingly, the coherent trend was observed only at 48 h in the root tissue for *miR167, miR403* and *miR426* and their respective targets. The transcript of *NtGT5b* (target of *miR398*) was constantly decreased in both tissues with 2- to 10-fold under drought conditions where the minimum peak was at 48 h and 24 h after treatment in root and leaf tissue, respectively. The expression of *(R)-mandelonitrile lyase* (target of *miR842*) increased at 12 h and decreased at subsequent time points in leaf tissue whereas it showed opposite pattern in root. In general, most targets exhibited their highest expression level at 48 h after stress depending on the tissue (Table 2; Figure 1).

### Expression of miRNAs and putative targets under heat stress

**Figure 2:**
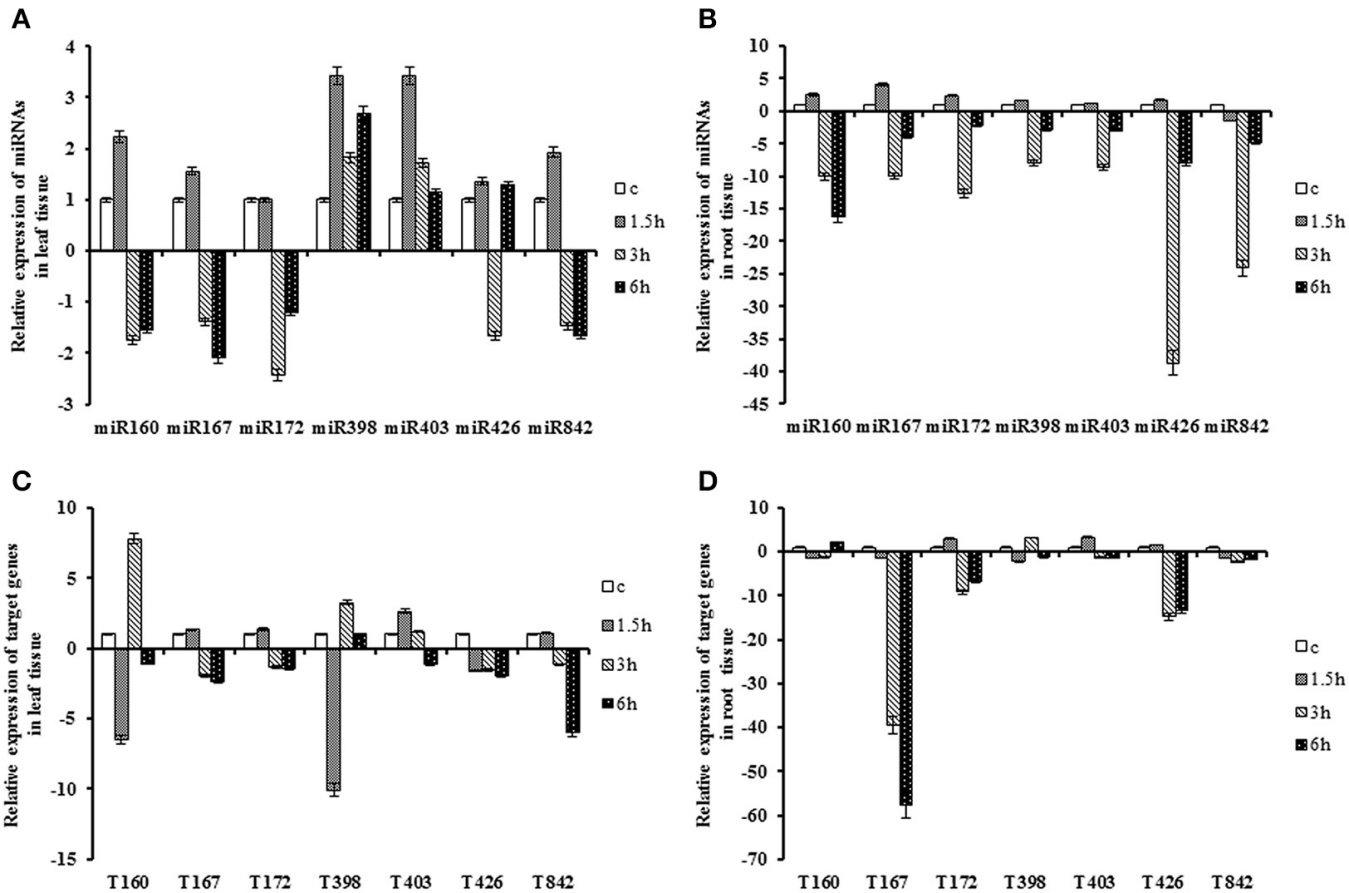
**Expression patterns of miRNAs and their target genes under heat stress**. Plants treated at 42 ± 1°C for 1.5, 3, and 6 h. **(A)** Expression patterns of miRNAs in the leaf. **(B)** Expression patterns of miRNAs in the root. **(C)** Expression patterns of target gene in the leaf. **(D)** Expression patterns of target genes in the root.

The expression levels of all seven miRNAs in leaf tissue were slightly up-regulated after 1.5 h exposure to heat stress, except for *miR172* which had constant expression level. However, expression of miRNAs in leaves indicated a mixed pattern at 3 and 6 h after treatment. The expression levels of *miR167* and *miR172* were down-regulated within a range of 2- to 3-fold at 3 and 6 h after stress. But in root tissues, their reduction was between 2 and 12 fold at these two time points. *MiR398* and *miR403* showed similar trend in leaf and root tissues. Their expression was immediately up-regulated in leaf at the initial time point, although, their expressions were decreased at 3 and 6 h after stress. The levels of their expression were higher than control condition. However, in root tissue, they were up-regulated at 1.5 h but down-regulated subsequently compared to control with a 2- to 8-fold change at 3 and 6 h. The *miR160, miR426* and *miR842* exhibited similar trend under heat stress in leaf tissue. Their expressions were induced at 1.5 h and down-regulated at 3 h and again induced at severe stress. Nevertheless, their expressions were lower compared to the control. In root tissue, except 1.5 h after stress, *miR842* and *miR426* showed similar pattern within a range of 4- to 38-fold change after stress. The expression of *miR160* instantly decreased at 3 h (10-fold change compared to control) and its decrease continued constantly at 6 h (Table 2; Figure 2).

The expression of *QHG18J04.yg.ab1* was significantly down-regulated at 1.5 h and was reversed at 3 h, while its expression was only up-regulated at 6 h in root. The pattern of its transcript had negative correlation with *miR160*. Transcript of *ARF6* was down-regulated at all-time points with a sharp decrease in the root tissue at 3 h. The temporal variation of *COX5b* was significant only in the root tissue while it decreased sharply at 3 h after heat stress. *AGO2* expressed at a significant level at 1.5 h in both tissues where its expression was up-regulated with more than two-fold change, compared to the control. *NtGT5b* displayed similar pattern in both tissues. The expression level declined at an initial time point and was induced at 3 h after treatment, whereas it showed coherent type with *miR398*. In leaves, the expression of *(R)-mandelonitrile lyase* was abruptly down-regulated at 6 h, whereas its expression was declined slightly over time in the root. Expression of *TPP2* dropped in leaf tissue, but it decreased in root only at 3 h with 14-fold changes after stress. However, there was a slight increase in its expression at 6 h in the root. The expression was still lower compared with control (Table 2; Figure 2).

### Expression of miRNAs and putative targets under salt stress

**Figure 3:**
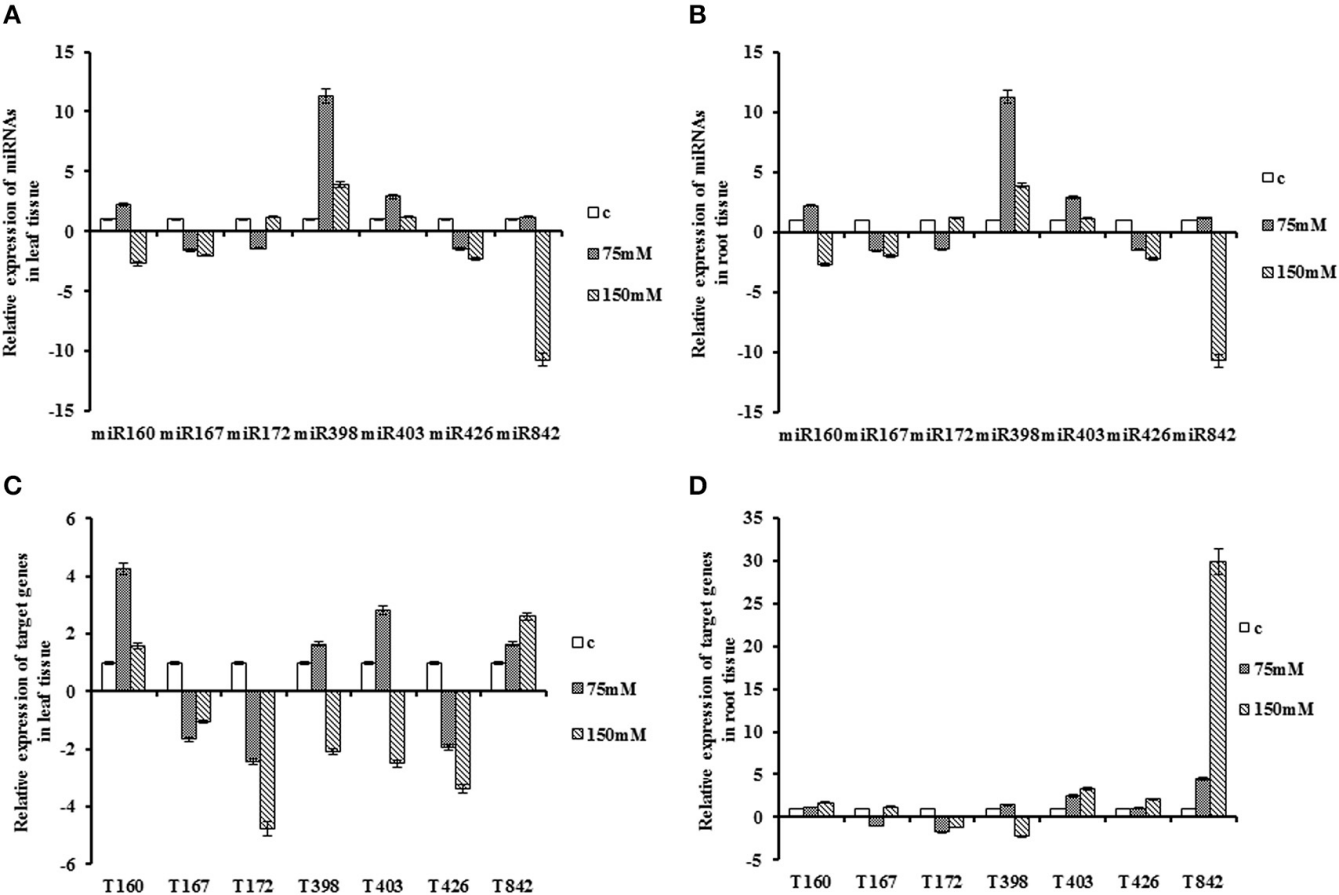
**Expression patterns of miRNAs and their target genes under salt stress**. Plant subjected under NaCl treatment in two concentration, 75 and 150 mM. **(A)** Expression patterns of miRNAs in the leaf **(B)** Expression patterns of miRNAs in the root. **(C)** Expression patterns of target gene in the leaf. **(D)** Expression patterns of target genes in the root.

The expression of *miR167* showed opposite pattern in both tissues. In leaves, salt stress reduced the expression of *miR167* by two-fold while the expression level was up-regulated in the root tissues with a two-fold change. Interestingly, *miR403* exhibited opposite pattern of expression in leaf and root, whereas its expression had increasing and declining trend in leaf and root, respectively. The expression trends of *miR160, miR426* and *miR842* were similar in both tissues at severe stress. Their expression showed decreasing pattern in leaf and increasing pattern in root tissue. Interestingly, an abrupt gradient was observed for *miR842* in leaf tissue at 150 mM NaCl. None of the tissues disclosed a significant alteration for *miR172* expression in response to salt stress. Salt stress induced the *miR398* expression in both tissues with the sharp increase observed after 75 mM NaCl treatment in both tissues. In spite of this, there was a considerable reduction in its expression at 150 mM concentration in leaf and root, and the expression level was higher than control condition (Table 2; Figure 3).

Under salt stress, the transcripts of *QHG18J04.yg.ab1* slightly increased in the root. Although, there was a decline pattern at 150 mM of NaCl in comparison with an early stage of stress in the leaf tissue, its expression was higher compared to control. The expression of *ARF6* was not significant in the root. However, its expression showed down-regulation at both stages of treatment in leaf tissue. Its expression level revealed a slightly increasing trend at 150 mM compared with 75 mM of NaCl. *COX5b* expression was lower in both tissues at all stages of treatment with sharp reduction at 150 mM of NaCl in the leaf tissue. *NtGT5b* exhibited similar expression pattern in both tissues where its mRNA level was up-regulated at 75 mM concentration and was abruptly decreased at 150 mM. The *AGO2* transcript was highly accumulated at all stages in the root tissue, but in the leaf tissue, was induced at 75 mM of NaCl, and down-regulated subsequently compared with control. The expression pattern of *TPP2* was opposite in leaf and root tissue, as decreased in leaf and increased in root tissue with its peak of expression at severe stress. The expression of *(R)-mandelonitrile lyase* was induced in both tissues with its peak at 150 mM concentration (Table 2; Figure 3).

### Expression of miRNAs and putative targets under cadmium stress

**Figure 4:**
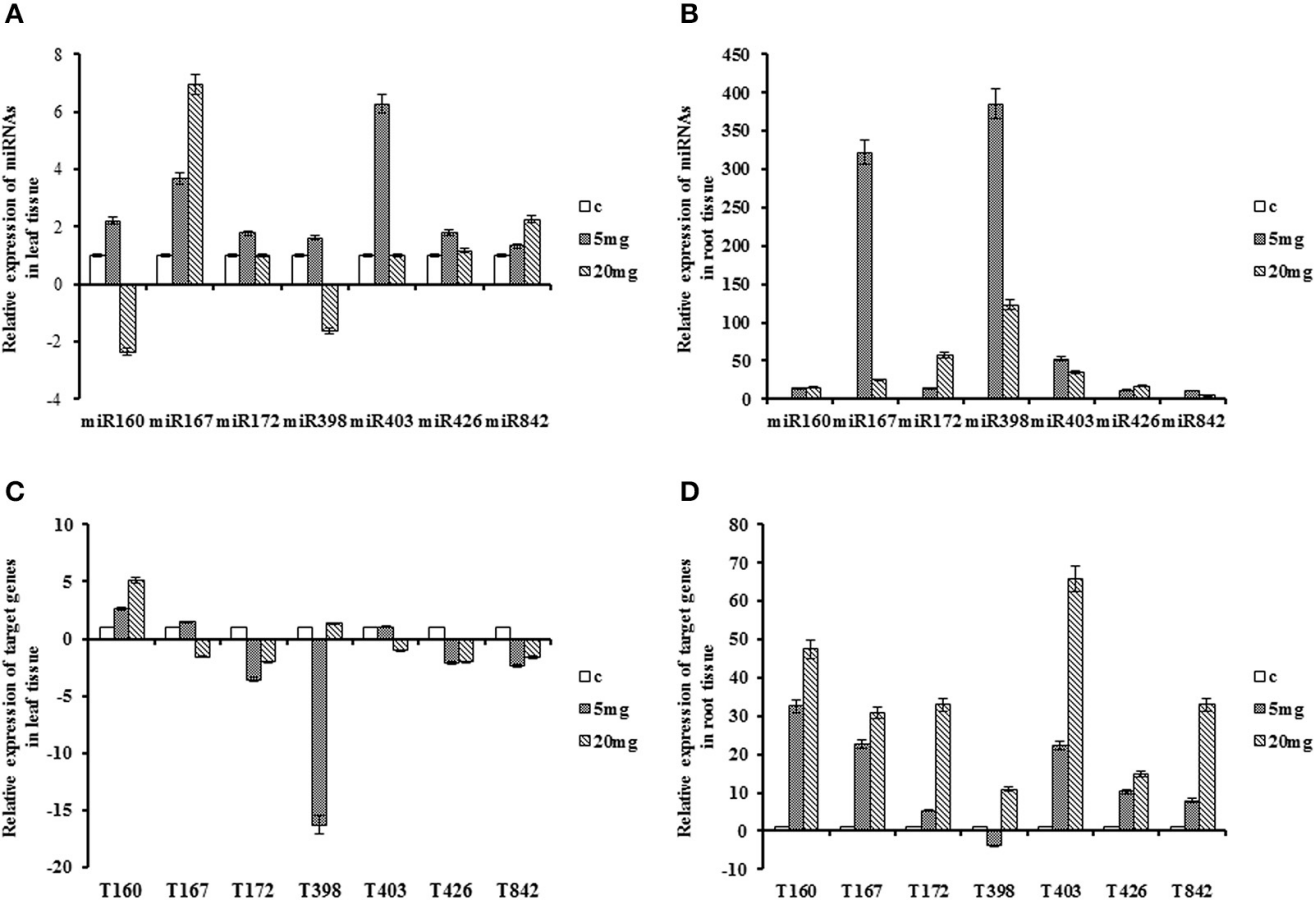
**Expression patterns of miRNAs and their target genes under cadmium stress**. Plants treated with cadmium in 5 and 20 mg/L concentration. **(A)** Expression patterns of miRNAs in the leaf. **(B)** Expression patterns of miRNAs in the root. **(C)** Expression patterns of target gene in the leaf. **(D)** Expression patterns of target genes in the root.

Cadmium treatment resulted in up-regulation of all miRNAs in roots, with fold-changes between 4 and 385. The lowest and highest peaks were for *miR842* in 20 mg/L and *miR398* in 5 mg/L concentration of cadmium, respectively. The expression of *miR426* was induced only at 5 mg/L in leaf, while in root induced at all stages of stress. The opposite pattern for *miR160* and *miR842* observed only at 20 mg/L in leaf tissue. The level of *miR160* was drastically decreased in leaf tissue, but the expression of *miR842* was slightly raised. In root tissue, their expression was increased although; the levels of *miR842* at 20 mg/L were declined in comparison to 5 mg/L, where its expression was still higher compared to control. *MiR403* expression was increased in both tissues whereas expression levels in root tissue were higher than leaf tissue. The highest expression of *miR403* was in 5 mg/L concentration with 52-fold change compared to control. The expression pattern of *miR172* was induced at all stages of treatment in both tissues with its peak at 20 mg/L concentration in root. Interestingly, the level of *miR398* was up-regulated at 5 mg/L, but down-regulated at 20 mg/L with 1.6-fold change in leaf tissue. Similar trend was observed for *miR167* and *miR398* in root tissue, with the highest peak at 5 mg/L concentration (Table 2; Figure 4).

Transcript of *QHG18J04.yg.ab1* showed up-regulation trend in both tissues where in root, it was over 10-fold higher than leaf. The target of *miR167* and *miR403* exhibited similar trend in both tissues as their expressions were significantly induced in the root with their highest peak at 20 mg/L of CdCl_2_. The expression of *COX5b* and *TPP2* was declined in leaf and was increased in root tissue in both concentrations of cadmium with its peak in 20 mg/L. *(R)-mandelonitrile lyase* highly accumulated in the root with abrupt increase at 20 mg/L. In contrast, its expression was decreased in leaf tissue after treatment. Expression of *NtGT5b* revealed temporal variation where it was down-regulated at 5 mg/L and up-regulated at 20 mg/L in both tissues. Interestingly, *miR172, miR398, miR426* and *miR842* showed inverse correlation with their targets in the leaf tissue at 5 mg/L (Table 2; Figure 4).

### Cluster analysis of miRNAs in *H. annuus* root and leaf tissues based on their expression patterns

**Figure 5:**
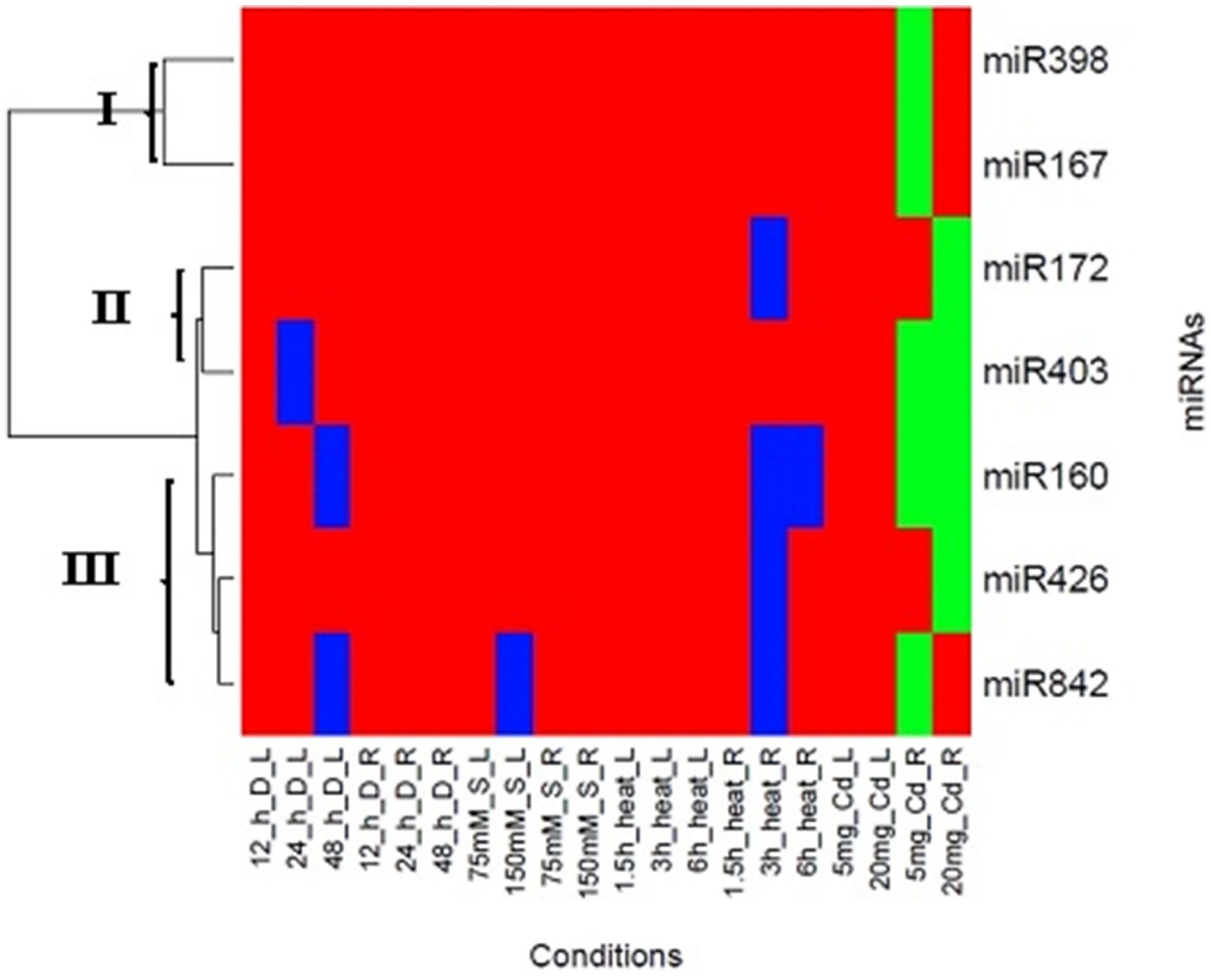
**Clustering of miRNAs expression profiles**. Heat map diagram of miRNA expression prepared with two-way unsupervised hierarchical clustering of miRNAs expression under different abiotic stress. miRNAs are given in the rows and each columns represent a sample. The miRNA clustering tree is shown on the left (cluster I, II and III). Abbreviations: L, Leaf; R, Root; h, Hour; D, Drought stress; S, Salt Stress; Cd, Cadmium stress.

Cluster analysis was performed to further analyze the pattern of expression of these miRNAs under different stress conditions. A comparison of expression profiles of these seven miRNAs in both tissues was conducted to find their tissue-specific expression patterns, regardless of their response to different stress conditions. Three clusters were formed, when the expression pattern of the seven miRNAs were analyzed in response to stress (Figure 5). Cluster I included *miR167* and *miR398*. The expression pattern of leaf and root was opposite, but their trend was similar in each tissue over time and stress. In root tissue, these miRNAs revealed slight alteration in three treatments; the highest peak was in 5 mg/L cadmium stress. Cluster II contained *miR172* and *miR403* families and showed a fluctuated pattern in leaves and roots in all of stress conditions. Median value of expression of these miRNAs exhibited increasing pattern in four stresses. Due to similarity in expression patterns of *miR160, miR426* and *miR842*, they grouped in the cluster III. They displayed similar trend in each tissue during stress. The lowest peak of *miR842* was in severe stress through drought and salt condition in the leaf tissue, while it was at 3 h after heat treatment in the root tissue.

### Relationship of miRNAs and target gene in stress response

Analysis of Pearson correlation showed positive and negative correlation between expression of miRNAs and target genes against stress in both tissues. The results indicated significant negative correlation between *miR172, miR398* and *miR403* and their putative targets in leaf tissue under cadmium stress. On the contrary, *miR398* presented significant negative correlation in root tissue after heat treatment. Weak correlation was observed between some miRNAs and their candidate targets in both tissues against specific stress such as *miR160* in drought stress and *miR167* after heat treatment (Table S4). Some miRNAs displayed significantly positive correlation, which indicated other mechanisms are involved in target gene regulation.

### Interaction between miRNAs and TFs-mediated gene regulatory subnetworks

**Figure 6:**
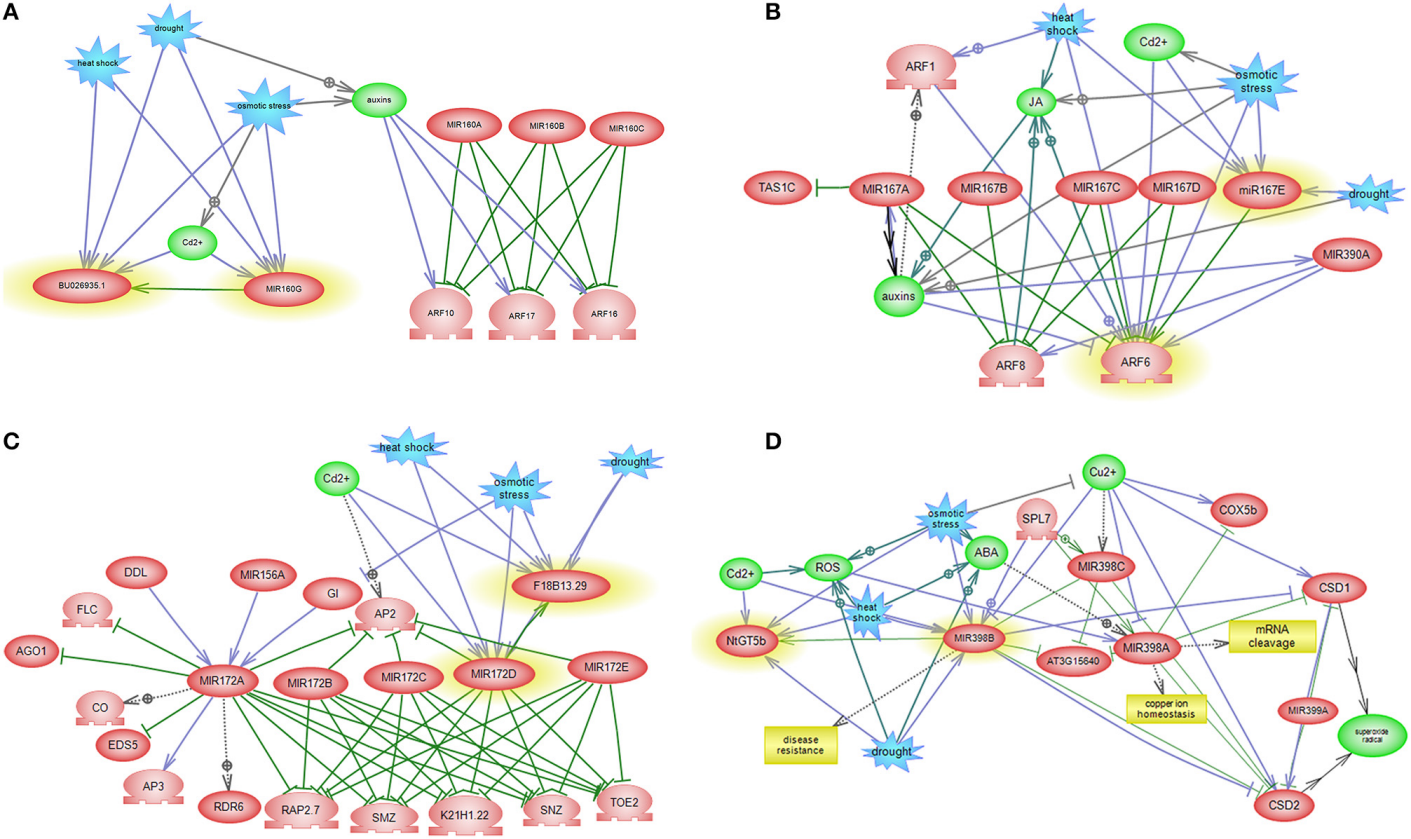
**miRNA-mediated gene regulatory sub networks in response to abiotic stress**. miRNA-target-abiotic stress interaction are shown. **(A)** Feedback loop between miR160, its target and abiotic stress. **(B)** Feedback loop between miR167, its target and abiotic stress and abiotic stress. **(C)** Feedback loop between miR172, its target and abiotic stress. **(D)** Feedback loop between miR398, its target and abiotic stress.

**Figure 7:**
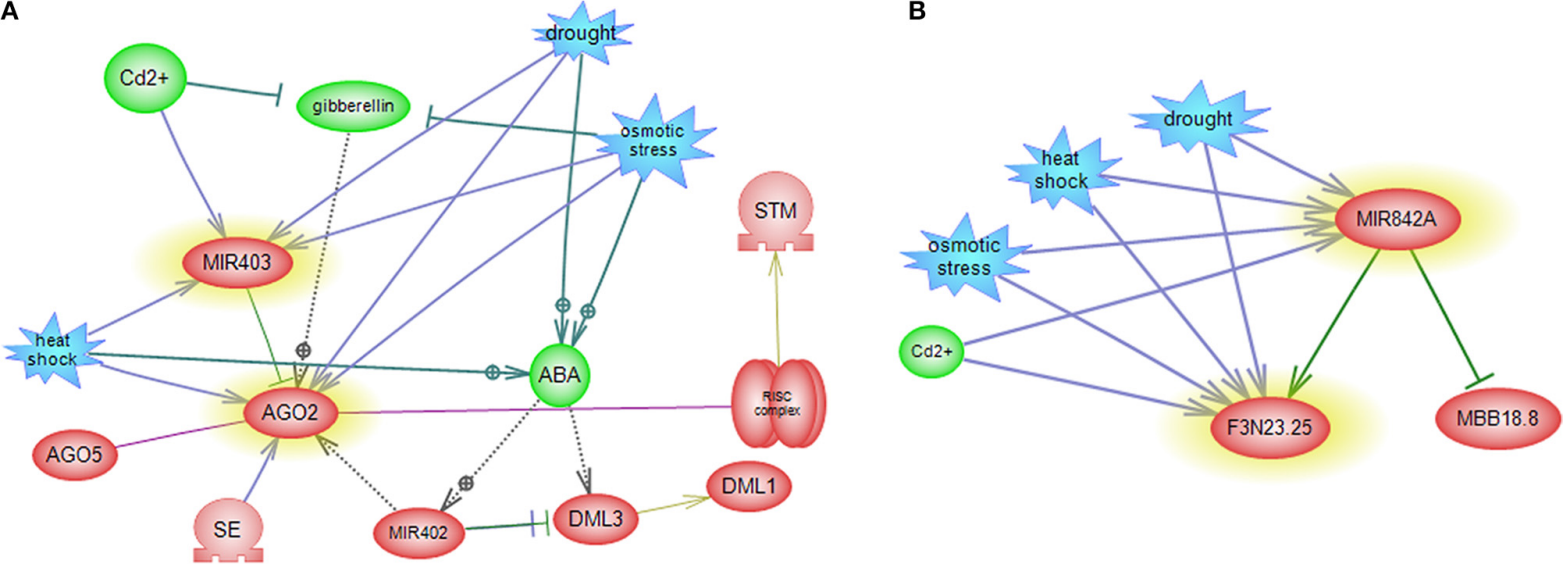
**miRNA-mediated gene regulatory sub networks in response to abiotic stress**. miRNA-target-abiotic stress interaction are shown. **(A)** Feedback loop between miR403, its target and abiotic stress. **(B)** Feedback loop between miR842, its target and abiotic stress.

The regulatory subnetworks that are constructed for each miRNA elucidated some of the intermingled miRNA and TF relationships as well as miRNA-miRNA relationships and the involvement of other miRNA families in miRNA specific post-transcriptional regulation pathways (Figures 6, 7). References of interaction relations between miRNAs and genes in subnetworks and their correlated references are listed in Table S5.

GO analysis is a robust approach in understanding underlying molecular mechanisms of different cellular events and offer a reliable tool for GO based gene selection (Fruzangohar et al., 2013). GO classification showed that the target genes are involved in auxin signaling, RNA mediated silencing, ethylene signaling, DNA methylation and response to abiotic stress.

Subnetwork of *miR426* did not display any connectivity with other participants in the network. As *miR426* exhibits stress and tissue specific pattern, this miRNA is a suitable candidate for further studies in the context of miRNA mediated gene regulatory pathway. *MiR842* showed connectivity with *MBB18_8* and *(R)-mandelonitrile lyase* that drought, heat, salt and cadmium affected its expressions (Figure 7B).

*MiR160* centered network exposed interaction between *QHG18J04.yg.ab1* and three TFs belonging to the *ARF* family (*ARF16, ARF17*, and *ARF18*), where four abiotic stress affect expression of *miR160* and *QHG18J04.yg.ab1* (Figure 6A). Interestingly, in this network, two other *ARF* family members; *ARF6* and *ARF8* regulate JA (Jasmonic acid) pathway and are in turn regulated by *miR167*. In addition, sub network of *miR167* includes regulation of *TAS1C*, which encodes a ta-siRNA (Figure 6B). Expression of TFs involved in ethylene signaling pathway and flower and seed development is regulated by the members of the *miR172* family. But *miR172* itself is regulated by *miR156* and *DDL* (a gene silencer) (Figure 6C). *MiR398* revealed cross talk with *SPL*, with central roles in many cellular processes such as auxin metabolism, root growth and cytokinesis; where *SPL* is regulated by *miR156, miR157* and *miR159*. Therefore, these three miRNAs are speculated to regulate the expression of *miR398* indirectly. *MiR156* have excelled as a key regulator in the regulation of other miRNAs. *MiR398* causes mRNA cleavage and disease resistance, which regulates expression of *CSDs* gene and *NtGT5b* (Figure 6D). Subnetwork of *miR403* revealed that *SE* and *miR402* regulate *AGO2*. It is known that *SE* has role in miRNA biogenesis, RNA splicing and chromatin modification. *MiR402* displayed connectivity with *DML1* and *DML3*, which are involved in DNA methylation and transcriptional control (Figure 7A).

## Discussion

The role of seven miRNAs in response to several abiotic stresses was studied in *H. annuus* leaf and root tissues by RT-qPCR. Changes in RWC, expression of *HSP70*-related protein, sodium, potassium, and cadmium concentration revealed obviously various mechanisms stimulation to cope with stress. The measured alterations of metabolic and physiological status in *H. annuus* provide additional support for modification reactions toward stress conditions.

The temporal and spatial expression profiles of seven miRNAs in both tissues at altered time points were in agreement with previous studies (Sunkar et al., 2012; Zandkarimi et al., 2015; Zhang, 2015). For instance, high-throughput sequencing of *Brassica napus* showed up-regulation of *miR172* in the root tissue under cadmium excess, up-regulation of *miR167* from 2 to 24 h after exposure in 300 mM NaCl in *Arabidopsis* and down-regulation of *miR398* under drought stress in cotton which treated with PEG.

The different expression patterns of *miR167* observed in photosynthetic and non-photosynthetic tissues suggest that *miR167* may have a role in tissue-specific adaptation to stress. *MiR160* and *miR167* regulate *ARF* families and play roles in the auxin signaling pathway (Khraiwesh et al., 2012), and were differentially expressed under stresses compared to control conditions. In addition, ARF domain was found in the protein sequence of *QHG18J04.yg.ab1* (target of *miR160*). Therefore, this gene may be involved in auxin signaling pathway and have a role in promoting plant tolerance to stress. The alteration of *miR160* and *miR167* in *H. annuus* at early stage of treatments suggests that they are responsive to early stress response. Reduced expression of *miR160* and *miR167* in leaves under severe stress may alter basal level of auxin and consequently restrict plant growth through the antagonism between abscisic acid and auxin because of escape of tension. In turn, this could lead to attenuation of plant growth and development under stress to cope with the imposed stress (Sunkar et al., 2012). Even though they have common targets, in our study, *miR160* and *miR167* did not group together in one cluster. On the other hand, these miRNAs showed various expression patterns in other plant species under different stress (Khraiwesh et al., 2012; Sunkar et al., 2012). Besides, under normal conditions, their expression pattern was also variable in different plant genus and species (Zeng et al., 2010).

Some studies have suggested roles for *miR172* during cadmium, drought, cold and heat stress conditions (Sunkar et al., 2012; Zhou et al., 2012). In line with the previous studies, *miR172* revealed temporal up- or down-regulation in leaf and root in response to stress conditions except for the salt stress. Expression of *miR172* showed inverse correlation with its target *COX5b*, only in leaf tissue under cadmium stress. The post-transcriptional silencing of *COX5b* by *miR172* may reflect the providence of *Helianthus* plant from energy loss via avoiding respiration under excessive cadmium concentrations (Sunkar et al., 2012).

We found that *miR398* is especially up-regulated in leaf tissues during heat stress in line with the previous reports (Guan et al., 2013). Interestingly, *miR398* was down-regulated in root tissue while expression of *HSP70*-related was up-regulated, which may indicate that *miR398* has a tissue specific mode of action and localization during heat stress condition. Differential expression of *miR398* in response to drought, salt, heat and cadmium stress have been shown in several species such as wild emmer wheat, *Medicago, Nicotiana, Brassica* and *Arabidopsis* (Trindade et al., 2010; Frazier et al., 2011; Kantar et al., 2011; Zhou et al., 2012; Guan et al., 2013). In contrast, under severe drought and cadmium stress conditions, *miR398* was down-regulated in leaf tissue indicating a tissue-specific and stress-specific response orchestrated by this miRNA. In this study, the probable target of *miR398* was *NtGT5b* which is a microsomal enzyme responsible for glucuronidation reactions with a role in the storage of secondary metabolites and plants defense against stress (Miners et al., 2002; Ko et al., 2006). On the other hand, new target genes were predicted for *miR398* and *miR167* in *Phaseolus vulgaris* and *Malus hupehensis* in vegetative phase which involved in monogalactosyl diacylglycerol synthase, acyltransferase and dioxygenase, gluconeogenesis pathway and glycolytic process (Heyndrickx and Vandepoele, 2012; Han et al., 2014; Xing et al., 2014). Furthermore, in *Nicotiana tabacum* in response to TiO2 nanoparticles, these miRNAs were grouped in one cluster (Frazier et al., 2014). All of these results have led to the conclusion that *miR398* may be involved in the sugar biosynthesis pathway, associated with reduction in energy consumption for photosynthesis, and increase the tolerance of plant in abiotic stress conditions.

Surprisingly, the pattern of *miR403* was varying in each stress condition. Its expression was declined and was induced in both tissues in drought and cadmium stress, respectively. In contrast, *miR403* displayed increased expression in the leaf and decreased expression in the root during heat and salt stress. In the previous study, abundance of *miR403* was high in heat and salt libraries in *Raphanus sativus* (Wang et al., 2014; Sun et al., 2015). As a result, this discrepancy suggested that *miR403* was potentially expressed in stress-, tissue-, and species- specific manner during abiotic stress. Also, the expression of *AGO2* at all stages of treatment in both tissues was aberrant. In plants, *AGOs* are involved in various small RNA pathways from post-transcriptional gene silencing to epigenetic silencing phenomena such as RNA-directed DNA methylation (RdDM) pathway in *Arabidopsis* (Schraivogel and Meister, 2014). AGO1 and AGO2 proteins were regulated by *miR403* (Figure 7A). In addition, AGO2 has been shown to have an antiviral role (Harvey et al., 2011). Therefore, it is possible that this miRNA is involved in the regulation of miRNA-mediated RNA cleavage carried out by other miRNAs during stress conditions. Furthermore, *AGO1* is also regulated by *miR172* family (Ronemus et al., 2006), which may designate a crucial role for *miR172* in general miRNA mediated gene silencing pathways (Figure 6C). This result, based on altered expression of *miR403* and its target under different abiotic stress and its subnetwork (Figure 7A) which showed *DML1* and *DML3*, involved in DNA methylation, may pave the ways for intricate control mechanisms for drought, heat, salt and cadmium stress tolerance in sunflower. Interestingly, *miR172* and *miR403* were grouped in one cluster (Figure 5) and they showed common targets; *AGO1* and *AGO2*, which may suggest a general role for these miRNAs in small RNA pathway and DNA methylation.

In this study, *miR426* and *miR842* show differential expression under abiotic stress. However, their expression exhibited stress-dependent manner during stress which was declined in root tissue under heat and drought stress and was reversed in salt and cadmium treatment. Also, inverse correlation of these miRNAs with their target was temporary in some stages. Their possible targets, *TPP2* and *(R)-mandelonitrile lyase*, were induced in response to some abiotic stresses and are reported to be involved in defense mechanism, oxidation-reduction process, cyanide biosynthetic process and alcohol metabolic process, which is indicated as a biocatalyst in organic chemistry (Hu and Poulton, 1999; Shima et al., 2007). *MiR842* revealed no significant change after waterlogging conditions (Moldovan et al., 2010), whereas it was repressed after ABA treatment of roots in *Arabidopsis* (Jia and Rock, 2013) which was similar with the expression of *miR842* under drought and heat stress in both tissues. In the earlier studies, *MBB18_8* a member of *Jacalin lectin* family (Gustafson et al., 2005) and a kinase-like protein (Barozai et al., 2012) were predicted as targets of *miR842*. As a result, *miR842* might have a role not only in sugar biosynthesis and sugar mediated signaling pathways but may also have a role as an osmoprotectant. Surprisingly, *miR842* gene was post-transcriptionally regulated by alternative splicing (Jia and Rock, 2013). Consequently, temporal expression in response to stress and regulation of its gene revealed that *miR842* might have a complicated function in plants. Our results suggest a novel function for *miR426* and *miR842* in the regulation of sunflower tolerance to abiotic stress. Interestingly, *miR160, miR426* and *miR842* showed similar pattern and were grouped in one cluster and according to their target, they are probably involved in carbohydrate signaling pathway.

In this study, expressions of miRNA targets are not consistent with the expression pattern of their related miRNAs at all times. The pattern of miRNAs and their target genes were semi-coherent, coherent or non-coherent type during stresses in leaf and root tissues. For example, expression of *miR842* and its target in leaf tissue showed inverse correlation during mild drought and cadmium stress. A recent study has revealed, the expression pattern of four miRNAs and their target have a semi-coherent fashion under salt stress in the halophyte smooth cordgrass (Zandkarimi et al., 2015). In addition, we did not analyze protein levels of target genes during stress, therefore we can neither confirm nor reject that these genes are direct targets for these miRNAs. It is possible that these miRNAs and their targets are expressed in a non-overlapping manner and regulate their targets in different cells, as reported for *miR395* and *AST68* in *Arabidopsis* (Kawashima et al., 2009). The coherent correlation between miRNA and mRNA is still under debate (Li et al., 2012), while the non-coherent type implicated the post transcriptional regulation mechanism by miRNA-directed cleavage for target mRNAs (Zandkarimi et al., 2015). Taken together, this information suggests that miRNAs play a versatile role for plant's acclimation to stress conditions. The kinetics of miRNAs and target regulation over time and in different tissues against tension and compression stresses imply complicated physiological and genetic mechanisms in *H. annuus* in order to deal with and adapt to harsh environment. Indeed, aberrant expression of many miRNAs during stress revealed that they respond to environmental stresses in a miRNA-, stress-, and tissue-dependent manner. Nevertheless, differential expression of certain miRNA rely on the specific stress condition, even in the same plant species (Zhang, 2015). As a consequence, aberrant expression of these miRNAs may reflect synergistic activities at the biochemical, physiological and molecular levels such as auxin signaling and sugar response, and finally at the organismal level to attenuate plant growth and development under stress. Interaction between miRNA and target gene is more flexible because of regulation of a mRNA target gene by multiple miRNAs or on the contrary regulation of numerous mRNA target by individual miRNA (Zandkarimi et al., 2015). As well, alternative splicing regulates miRNA biogenesis and expression of target genes to make different isoforms (Jia and Rock, 2013). This indicates that plants employ unrecognized regulatory loops to achieve tolerance via these regulatory small RNAs and suggests that they selectively regulate the expression of specific target genes under each condition. In conclusion this study adds to the growing body of literature on stress-responsive miRNAs in plants.

## Author contributions

BS conceived and designed the research. BS, EE, HF, FR, and HB conducted experiment. RE, SM, FK, and ZF carried out experiment and analyzed data. RE, BS, HF, and EE wrote the manuscript. All authors read and approved the manuscript.

### Conflict of interest statement

The authors declare that the research was conducted in the absence of any commercial or financial relationships that could be construed as a potential conflict of interest.
