# Supplementary material for: Differential expression of seven conserved microRNAs in response to abiotic stress and their regulatory network in Helianthus annuus
Source: Front Plant Sci. 2015 Sep 17;6:741. doi: 10.3389/fpls.2015.00741 (PMC4585256; doi:10.3389/fpls.2015.00741)
Supplement: Supplementary file 1 [file TableS1-S4.DOC]

**Table S1. Primers used in this study**

| **miRNA name** | **miRNA forward primer** | **miRNA RT primer** |
| --- | --- | --- |
| Han-miR160 | TTGTGCCTG GCTCCCTGT | GTCGTATCCAGTGCAGGGTCCGAGGTATTCGCACTGGATACGACTGGCAT |
| Han-miR167 | GGCGGTTGAAGCTGCCAGCA | GTCGTATCCAGTGCAGGGTCCGAGGTATTCGCACTGGATACGACAGATCA |
| Han-miR172 | GCCGCCAGAATCCTGATGA | GTCGTATCCAGTGCAGGGTCCGAGGTATTCGCACTGGATACGACGCAGCA |
| Han-miR398 | GCCGCCGTGTTCTCAGGT | GTCGTATCCAGTGCAGGGTCCGAGGTATTCGCACTGGATACGACGGGGCG |
| Han-miR403 | GCCGCCTTAGATTCACGCACA | GTCGTATCCAGTGCAGGGTCCGAGGTATTCGCACTGGATACGACCGAGTT |
| Han-miR426 | GCCGCCCTTTGGAAGTTTGTC | GTCGTATCCAGTGCAGGGTCCGAGGTATTCGCACTGGATACGACACTAAG |
| Han-miR842 | GCCGCCTCATGGTCAGATTCA | GTCGTATCCAGTGCAGGGTCCGAGGTATTCGCACTGGATACGACGGATGA |

| **Primer name** | **5'………3'** | **Length**  **(bp)** | **Tm**  **(°C)** | **Product**  **length** | **Gene name** |
| --- | --- | --- | --- | --- | --- |
| Han-18S-F | TTCAGACTGTGAAACTGCGAATGG | 24 | 58 | 178 | 18S rRNA |
| Han-18S-R | TCATCGCAGCAACGGGCAAA | 20 | 62 |
| Han-QHG18J04.yg.ab1-F | ACCCGTTTTCAAGCAATCC | 19 | 59 | 126 | QHG18J04.yg.ab1 |
| Han-QHG18J04.yg.ab1-R | AGGAAAACGACCCAACTGAA | 20 | 59 |
| Han- ARF6-F | GTCTTGAAGGCCAACTGGAG | 20 | 59.99 | 149 | ARF6 |
| Han- ARF6-R | CTCTTGCGGCGACACTATTT | 20 | 60 |
| Han-COX5B-2-F | GCCGGAATAAGAATCTGGTG | 20 | 59.53 | 141 | COX5B-2 |
| Han-COX5B-2-R | AGCAATGGTGTACCACCTCA | 20 | 59.01 |
| Han- NtGT5b -F | GCCCTCAACAGCGTCTCTAC | 20 | 60 | 131 | NtGT5b |
| Han- NtGT5b -R | AAGGGCTGCAAACAATTCTC | 20 | 59 |
| Han- AGO2-F | GAGATGGGTCGAAGTGCCTA | 20 | 55.00 | 137 | AGO2 |
| Han- AGO2-R | CCAAGGTTTTATTCGACACACA | 22 | 40.91 |
| Han- TPP2-F | TCGTCACATTAGACCCTGGA | 20 | 59 | 139 | TPP2 |
| Han- TPP2-R | TTCGACACCACAGTTGAAGG | 20 | 59 |
| Han- (R)-mandelonitrile lyase -F | AGTGACTCTGCTGGTGTCCA | 20 | 59.44 | 141 | (R)-mandelonitrile lyase |
| Han- (R)-mandelonitrile lyase -R | GTTAGGCAAGCATCCACCAT | 20 | 59.96 |

**Changes in RWC, stress-related gene, sodium, potassium and cadmium concentrations in different tissues of plant grown under stress**

**Expression of stress-related gene**

HSP70-related protein, in root of sunflower after exposure to heat stress was up regulated under three times of stress and highest of its expression was more than 6-fold at 6h after stress (Table S2).

**Sodium Influx, Association with Potassium Uptake**

The levels of Na^+^ and Na^+^/K^+^ were increased in both tissues during salt stress which high content of them was measured at concentration of 150mM (Table S3).The K^+^ content was significantly reduced in both leaves and roots (P <0.05), whereas reduction of it was notable at concentration of 75mM NaCl in root tissue (Table S3).

**Cadmium Concentration**:

Accumulation of cadmium showed in both tissues after stress which it in root was higher than leaf tissue. Expectedly, highest increase in cadmium contents was measured for plant treated with 20 mg of cadmium, where this increases was seventeen and sixteen times in leaf and root compared to control, respectively (Table S3).

**Measuring the RWC**

Leaf water status was monitored by measuring the plant RWC. The RWC around 82% in control plants while it was in mild, moderate and severe stresses 68%, 61% and 34% respectively (Table S3).

**Table S2. Expression pattern of HSP70-related protein in root of sunflower after heat stress. Plants grew on hydroponic under normal condition for two weeks and then leaves and roots of plants were harvested as control. Plants were treated at 42̊C for 1.5, 3 and 6 h and roots harvested for expression pattern of HSP70-related protein analysis (Gene Bank ID: AAB57695.1).**

| **Time** | **relative expression of HSP70-related protein to control** |
| --- | --- |
| C | 1 |
| 1.5h | ↑2.31** |
| 3h | ↑1.76** |
| 6h | ↑6.96** |

**Table S3. a) Salt tolerance assay and cadmium concentration in leaves and roots of sunflower (*Helianthus annuus* L.). Accumulation Na+, K+ and Na+/K+ ratio in control plants and treated plants with 75 and 150 mM NaCl. Data are means ± *SE*; *P* < 0.05. Plants treated with 5 and 20 mg/L with respect to controls were significantly statistic at *P* ≤ 0.05.**

**b) Measuring the RWC in leaf tissue drought under stress.**

**a)**

| **concentration** | | **Tissue** | |
| --- | --- | --- | --- |
|  | | **Leaf** | **Root** |
| Na+ | C | 0.027 | 0.038 |
|  | 75mM | 0.056^*^ | 0.093^*^ |
|  | 150mM | 0.0763^*^ | 0.1256^*^ |
| K+ | C | 1.987 | 3.579 |
|  | 75mM | 1.069^*^ | 2.999^*^ |
|  | 150mM | 0.993^*^ | 3.0416^*^ |
| K+/Na+ | C | 73.67 | 94.48 |
|  | 75mM | 19.27^*^ | 31.31^*^ |
|  | 150mM | 13.02^*^ | 24.63^*^ |
| Cd | C | 0.3 | 0.37 |
|  | 5mg/L | 0.51^*^ | 1.07^*^ |
|  | 20mg/L | 5.33^*^ | 6.25^*^ |

**b)**

| **Time of stress** | **Measuring the RWC** |
| --- | --- |
| Control | 82% |
| 12h (mild stress) | 68% |
| 24h (moderate stress) | 61% |
| 48h (severe stress) | 34% |

**Table S4. Pearson correlation between expression of miRNAs and target genes in leaf and root tis**

| **Number of observed negative relation between microRNA and target gen** | **Heat stress** | | **Drought stress** | | **Cadmium stress** | | **Salt stress** | |  |
| --- | --- | --- | --- | --- | --- | --- | --- | --- | --- |
|  | **Root** | **Leaf** | **Root** | **Leaf** | **Root** | **Leaf** | **Root** | **Leaf** | **microRNA** |
| **4** | -49.4 (P=0.05) | -47%  (NS) | 63.0% (p=0.05) | 67.1% (p=0.05) | -39.6%  (NS) | -20.0%  (NS) | 20%  (NS) | 87.5 %  (p =0.01) | **miR160** |
| **2** | 45.5% (P=0.1) | 59.8% (P=0.05) | 88.6% (p=0.01) | 81.3% (p=0.01) | 32.6%  (NS) | 40.0%-  (NS) | -12.2%  (NS) | 77.1%  (p =0.01) | **miR167** |
| **3** | 90.7%  (p =0.01) | 32.1% (NS) | -20.4%  (NS) | 86.9% (p=0.01) | 94.2%  (P=0.01) | 60.3%-  (P=0.05) | 71.4%  (p =0.01) | -17.2%  (NS) | **miR172** |
| **4** | -66.8% (P=0.01) | -21.8% (NS) | 57.4%  (P=0.05) | 89.1%  (P=0.01) | -24.2%  (NS) | -76.8%  (P=0.01) | 76.7%  (p =0.01) | 46.2%  (NS) | **miR398** |
| **2** | 69.5% (P=0.01) | 90.3% (P=0.01) | 36.6%  (NS) | 68.6%  (P=0.01) | 41.9%  (NS) | 76.8%-  (NS) | -66.5%  (P=0.05) | 82.4%  (P=0.01) | **miR403** |
| **2** | 92.9% (P=0.01) | -7.9%  (NS) | 16.9%  (NS) | 89.7% (P=0.01) | 93.3%  (P=0.01) | 47.9%-  (NS) | 35.1%  (NS) | 89.1%  (P=0.01) | **miR426** |
| **3** | 41.4% (NS) | 50.5% (P=0.05) | 72.0% (P=0.01) | -2.5%  (NS) | 04.9%  (NS) | 39.6%-  (NS) | 96.8%  (P=0.01) | -68.1%  (P=0.05) | **miR842** |
|  | **2** | **3** | **1** | **1** | **2** | **7** | **2** | **2** | **Number of observed negative relation between microRNA and target gen** |
